# Mortality prediction using CHADS_2_/CHA_2_DS_2_-VASc/R_2_CHADS_2_ scores in systolic heart failure patients with or without atrial fibrillation

**DOI:** 10.1097/MD.0000000000008338

**Published:** 2017-10-27

**Authors:** Yung-Lung Chen, Ching-Lan Cheng, Jin-Long Huang, Ning-I Yang, Heng-Chia Chang, Kuan-Cheng Chang, Shih-Hsien Sung, Kou-Gi Shyu, Chun-Chieh Wang, Wei-Hsian Yin, Jiunn-Lee Lin, Shyh-Ming Chen

**Affiliations:** a Section of Cardiology, Department of Internal Medicine, Kaohsiung Chang Gung Memorial Hospital, Kaohsiung City; b Chang Gung University College of Medicine; c Department of Pharmacy and Institute of Clinical Pharmacy and Pharmaceutical Sciences, College of Medicine, National Cheng Kung University; d Cardiovascular Center, Taichung Veterans General Hospital, Taichung; e Division of Cardiology, Department of Internal Medicine, Chang Gung Memorial Hospital, Keelung; f Division of Cardiology, Taipei Tzu Chi Hospital, Buddhist Tzu Chi Medical Foundation, New Taipei City; g Graduate Institute of Biomedical Sciences, China Medical University; h Division of Cardiovascular Medicine, China Medical University Hospital, Taichung; i Division of Cardiology, Taipei Veterans General Hospital; j Division of Cardiology, Shin Kong Wu Ho-Su Memorial Hospital, Taipei; k Division of Cardiology, Chang Gung Memorial Hospital, Linkou; l Heart Center, Cheng Hsin General Hospital; m Division of Cardiology, Department of Internal Medicine, National Taiwan University Hospital, Taipei, Taiwan, Republic of China.

**Keywords:** atrial fibrillation, mortality, risk score, systolic heart failure

## Abstract

The CHADS_2_, CHA_2_DS_2_-VASc, and R_2_CHADS_2_ scores are well-known predictors of stroke caused by atrial fibrillation (AF), but no studies have evaluated their use for stratifying all-cause mortality risk in patients discharged for systolic heart failure (SHF) with or without AF.

This study analyzed data in the Taiwan Society of Cardiology—heart failure with reduced ejection fraction (TSOC-HFrEF) registry. These data were obtained by a prospective, multicenter, observational survey of patients treated at 21 medical centers in Taiwan after hospitalization for acute, pre-existing or new onset SHF from May, 2013 to October, 2014. During 1 year follow-up, 198 patients were lost follow-up, and final 1311 (86.8%) patients were included for further analysis. During the follow-up period, 250 (19%) patients died. Multivariate analysis revealed that body mass index, thyroid disorder, valvular surgery history, chronic kidney disease (CKD), and scores for CHADS_2_, CHA_2_DS_2-_VASc, and R_2_CHADS_2_ were significant independent predictors of mortality in the overall population of SHF patients (all *P* < .05) The c-indexes showed that CHADS_2_, CHA_2_DS_2_-VASc, and R_2_CHADS_2_ scores were significantly associated with mortality in SHF patients with or without AF (all *P* < 005). However, R_2_CHADS_2_ had significantly higher accuracy in predicting mortality in all SHF patients compared with CHADS_2_ and CHA_2_DS_2_-VASc (DeLong test, *P* < .0001), especially in SHF without AF (DeLong test, *P* = .0003).

Scores for CHADS_2_, CHA_2_DS_2_-VASc, and R_2_CHADS_2_ can be used to predict 1-year all-cause mortality in SHF patients with or without AF. For predicting all-cause mortality in SHF patients, R_2_CHADS_2_ is more accurate than CHADS_2_ and CHA_2_DS_2_-VASc.

## Abstract 2

Supplemental Digital Content is available in the text

## 1 Introduction

Various risk scores currently used to predict mortality in systolic heart failure (SHF) patients have proven to be too complex for routine clinical use.^[1–5]^ The variables used in previous risk models have been very numerous and heterogeneous. Examples include clinical status, therapy (pharmacological and devices), laboratory parameters, and even functional test outcomes, which can change during hospitalization and with the progression of the disease. Some risk models are highly complex and may require laborious data entry or even the use of an interactive program.^[1,2,4,5]^ In patients with atrial fibrillation (AF), the most commonly used predictors of stroke scores are CHADS_2_ [congestive heart failure (CHF), hypertension, age, diabetes, stroke (doubled)], CHA_2_DS_2_-VASc [CHF, hypertension, age ≥75 (doubled), diabetes, stroke (doubled), vascular disease, Age 65–74, and sex category (female)], and R_2_CHADS_2_ [renal dysfunction (doubled), CHF, hypertension, age, diabetes, stroke (doubled)]. These scores are easily calculated because they include common cardiovascular risk factors^[6–10]^ and have been extended for use in predicting endpoints other than stroke^[11–13]^ and even for use in patients without AF.^[14–17]^ Some studies have shown that they can also be used to predict all-cause mortality in heart failure (HF) patients.^[17,18]^ However, their effectiveness for stratifying mortality risk in SHF patients with and without AF is unknown. Therefore, this study investigated the value of these risk scores for predicting all-cause mortality in patients with SHF.

## 2 Methods

### 2.1 Study designs and patients

The Taiwan Society of Cardiology—Heart Failure with reduced Ejection Fraction (TSOC-HFrEF) registry contains data obtained by a prospective, multicenter, observational survey of patients treated for HF at 21 medical centers in Taiwan. The inclusion criteria for the survey were age older than 18 years and hospitalization for either acute new-onset HF or acute decompensation of chronic HF with reduced left ventricular ejection fraction (LVEF). Enrolment criteria included LVEF less than 40% documented before enrollment by either echocardiography or left ventriculography during the index hospitalization. The only exclusion criterion was age less than 18 years old. The patients were consecutively enrolled at each participating site. Because this was an observational study, no specific protocol was established, and no recommendations for HF evaluation and management were made. Drug prescriptions, diagnostic tests, and therapeutic managements were left to the discretion of the attending cardiologists. Data were collected only after participating patients read the study information and gave written informed consent. Data collection for the index hospitalization included the period from the time of initial care to the time of discharge or death. Follow-up data were collected after 6 and 12 months. The design of this registry study was approved by the institutional review board (IRB) of each participating institution. Data were collected with a uniform case report form approved by the IRB of each medical center. After obtaining written informed consent to participate from each patient, the hospital investigator or research coordinator entered the patient data into an online database. Data collected from medical records included baseline characteristics, medical history, HF severity, echocardiographic data, in-hospital mortality, and discharge medications. The body mass index (BMI) and other echocardiographic data were also obtained. All other data were self-reported and confirmed by available medical records. The detailed study protocol is described in the previous report.^[19]^

### 2.2 Definition

Renal dysfunction and chronic kidney disease (CKD) were defined as an estimated glomerular filtration rate (eGFR) < 60 mL/min/1.73 m^2^. The eGFR was calculated using the abbreviated Modification of the Diet in Renal Disease Study equation: eGFR (mL/min/1.73 m^2^) = 186.3 × (serum creatinine [mg/dL])^−1.154^× (age [years])^−0.203^× (0.742 in females).^[20]^ Alcoholism were defined as patients whose average daily wine consumption exceeded 300 mL or whose average daily liquor consumption exceeded 60 mL.

### 2.3 Statistical analysis

The statistical analyses included all enrolled patients. Descriptive summaries were presented for all patients and for all subgroups of patients. Quantitative data were expressed as means ±  standard deviation; categorical variables were reported as percentages. Student *t* test was used to compare continuous data, and *χ*^2^ test or Fisher exact test was used to compare categorical data. A multivariate analysis was performed with a logistic stepwise regression model to determine the independent predictors of 1-year mortality. The CHADS_2_, CHA_2_DS_2_-VASc, and R_2_CHADS_2_ scores were sequentially entered in 3 different models (CHADS_2_ in model 1, CHA_2_DS_2_-VASc in model 2, and R_2_CHADS_2_ in model 3) for multivariate analysis. All the variables in Table 1, supplementary Table 1 and supplementary Table 2 with *P* value < .05 in predicting mortality by univariate analysis were enrolled into multivariate analysis except those variables of the different scoring systems in different models. The accuracies of CHADS_2_, CHA_2_DS_2_-VASc, and R_2_CHADS_2_ scores for predicting all-cause mortality were calculated by c-indexes based on receiver operating characteristic (ROC) curves. Areas under the ROC curves for these 3 scoring systems were compared using DeLong test. The net reclassification index (NRI) was used to quantify the accuracy of the risk scoring system in classifying subjects, as compared with other risk-scoring systems when the DeLong test revealed differences in area under the ROC curves for the 3 scoring systems. A *P* value of < .05 was considered statistically significant. The statistical analyses were performed using SAS statistical software Version 9.4 (SAS Institute, Cary, NC).

**Table 1:**
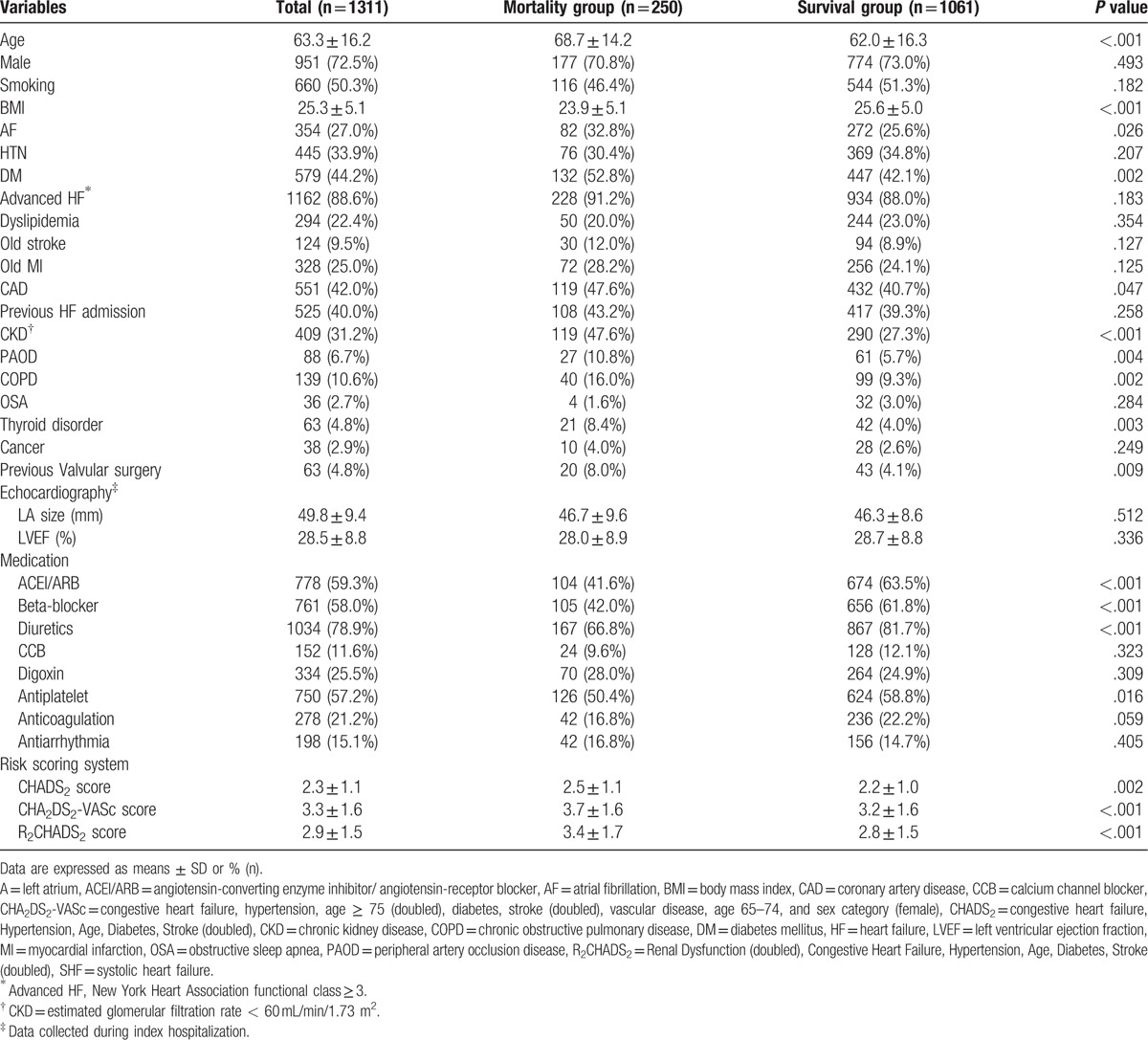
Baseline characteristics of 1-year mortality group and 1-year survival group in 1311 SHF patients.

## 3 Results

### 3.1 Baseline characteristics and 1-year mortality in SHF patients

The TSOC-HFrEF registry contains data for 1509 patients treated in 21 medical centers from May, 2013 to October, 2014. Detailed baseline characteristics are presented in our previous registry report.^[19]^ The TSOC-HFrEF registry contains data for an observational survey with no specific protocol for intervention during follow-up. Patients were free to withdraw their consent and participation for any reason and at any time. During the first year, 198 (13.2%) patients were lost to follow-up. Therefore, 1311 (86.8%) patients were included in the final analysis. Of these, 354 (27%) patients had a history of AF rhythm. During the 1-year follow-up, 250 (19%) patients died. Table 1 compares SHF patients who died (mortality group) and SHF patients who survived (survival group) during the 1-year follow-up. The mortality group was characterized by significantly older age (68.7 ± 14.2 vs 62.0 ± 16.3 years, *P* < .001), significantly lower BMI (23.9 ± 5.1 vs 25.6 ± 5.0, *P* < .001), and significantly higher incidences of the following: diabetes mellitus (DM) (52.8% vs 42.1%, *P* = .002), coronary artery disease (CAD) (47.6% vs 40.7%, *P* = .047), CKD (47.6% vs 27.3%, *P* < .001), peripheral artery occlusion disease (PAOD) (10.8% vs 5.7%, *P* = .004), chronic obstructive pulmonary disease (16% vs 9.3%, *P* = .002), thyroid disorder (8.4% vs 4%, *P* = .003) and previous valvular surgery (8% vs 4.1%, *P* = .009). Compared with the survival group, the mortality group also had significantly lower incidences of treatment with the following: beta-blocker, angiotensin-converting enzyme inhibitors/angiotensin-receptor blockers (ACEI/ARB), diuretics and antiplatelet (all *P* < .05). Finally, the mortality group had significantly higher scores for R_2_CHADS_2_, CHADS_2_, and CHA_2_DS_2_-VASc (3.4 ± 1.7 vs 2.8 ± 1.5, *P* < .001; 2.5 ± 1.1 vs 2.2 ± 1.0, *P* = .002; 3.7 ± 1.6 vs 3.2 ± 1.6, respectively, *P* < .001).

### 3.2 Multivariate analysis 1-year all-cause mortality predictors in SHF patients

Multivariate analysis of all variables with *P* value less than.05 showed that BMI, thyroid disorder, and valvular surgery history were independent predictors of 1-year mortality in all 3 models (all *P* < .05). CKD was a significant independent predictor of 1-year mortality in model 1 and model 2 (*P* < .001). The CHADS_2_ score (odds ratio 1.148, 95% CI: 1.003–1.315, *P* = .045), CHA_2_DS_2_-VASc score (odds ratio 1.130, 95% CI: 1.031–1.239, *P* = .009), and R_2_CHADS_2_ score (odds ratio 1.282, 95% CI: 1.172–1.402, *P* < .001) were independent predictors of 1-year mortality in the different models. Table 2 shows the results.

**Table 2:**
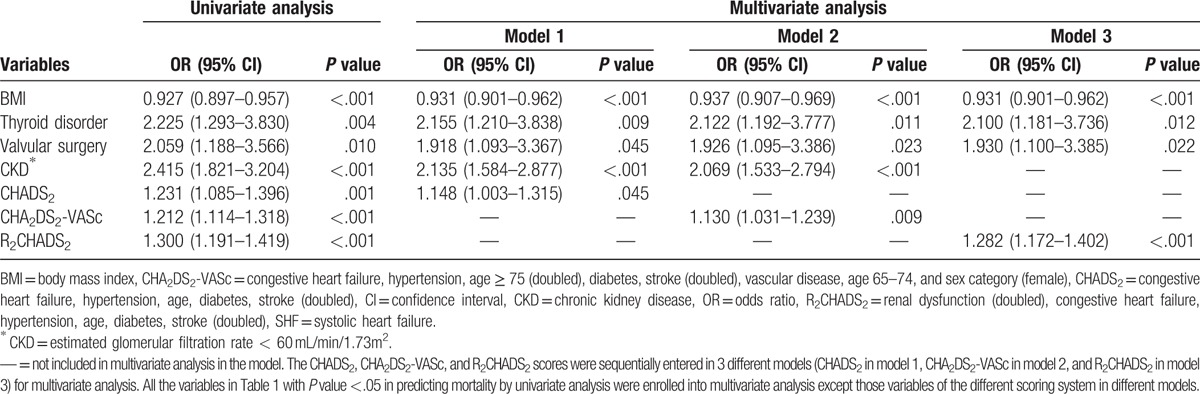
Univariate and multivariate analysis of 1-year mortality in SHF patients.

### 3.3 Baseline characteristics and 1-year mortality in total SHF patients with or without AF

Table 3 lists the baseline characteristics, in-hospital mortality, and 1-year mortality in SHF patients with or without AF. Of 354 SHF patients with a history of AF, 82 (23%) patients died during the 1-year follow-up. Supplemental Table 1 compares the mortality group and the survival group. Briefly, the mortality group were significantly older than the survival group (71.9 ± 13.1 vs 67.6 ± 14.5 years, respectively, *P* = .011) and were significantly more likely to have DM, CKD, previous valvular surgery, and paroxysmal AF (all *P* < .05). The survival group were also significantly more likely to receive treatment with beta-blocker, ACEI/ARB, and diuretics (all *P* < .05). The mortality group had significantly higher scores compared with the survival group for CHADS_2_ (2.7 ± 1.1 vs 2.3 ± 1.1, respectively, *P* = .011), for CHA_2_DS_2_-VASc (4.1 ± 1.5 vs 3.4 ± 1.7, respectively, *P* = .001), and for R_2_CHADS_2_ (3.7 ± 1.7 vs 2.9 ± 1.6, respectively, *P* < .001). Multivariate analysis in different models showed that valvular surgery history, CKD, CHADS_2_, CHA_2_DS_2_-VASc, and R_2_CHADS_2_ were independent predictors of 1-year mortality. Table 4 shows the statistical results. In patients who had SHF but not AF, 168 (17.6%) patients died during the 1-year follow-up. Supplemental Table 2 compares the mortality group and the survival group. Multivariate analysis in different models showed that independent predictors of 1-year morality were BMI, CKD, and R_2_CHADS_2_. Table 5 shows the statistical results. Comparisons of patients with and without CKD showed that the CKD group had more elderly people with low BMI. Supplemental Table 3 also shows that the CKD group had more patients with DM, CAD, PAOD, previous stroke, previous myocardial infarction, and previous hear failure admission.

**Table 3:**
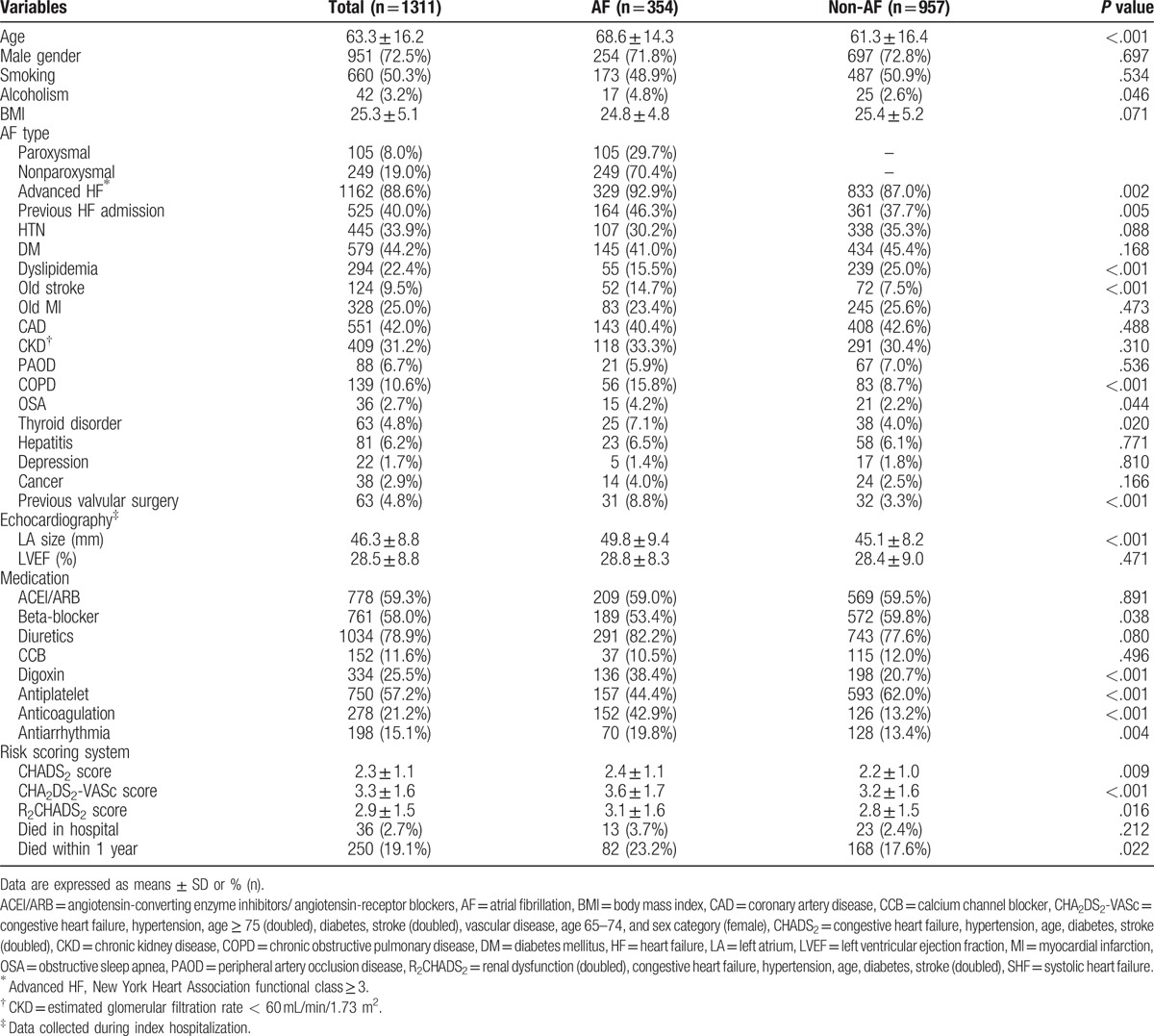
Baseline characteristics of SHF patients with and without AF.

**Table 4:**
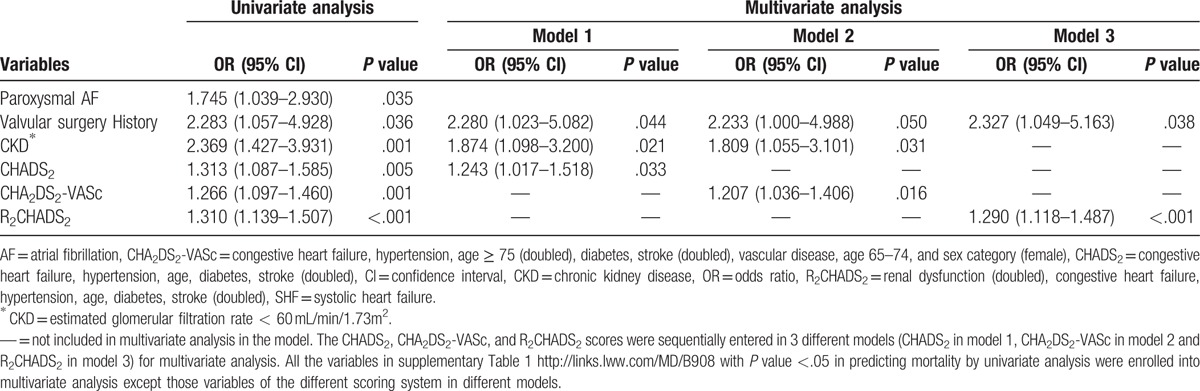
Univariate and multivariate analysis of 1-year mortality in SHF patients with AF.

**Table 5:**
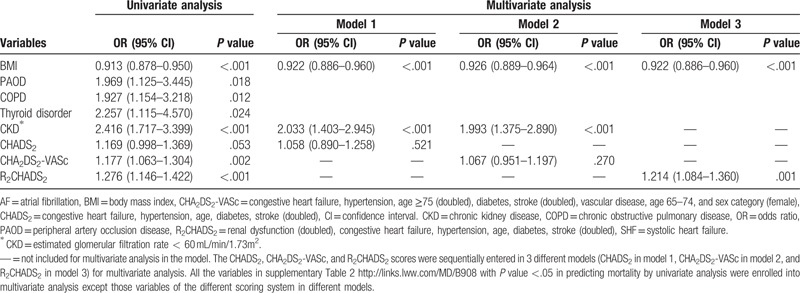
Univariate and multivariate analysis of 1 year mortality in SHF patients without AF.

### 3.4 Comparisons of different scoring systems as predictors of 1-year mortality in SHF patients with or without AF

**Figure 1:**
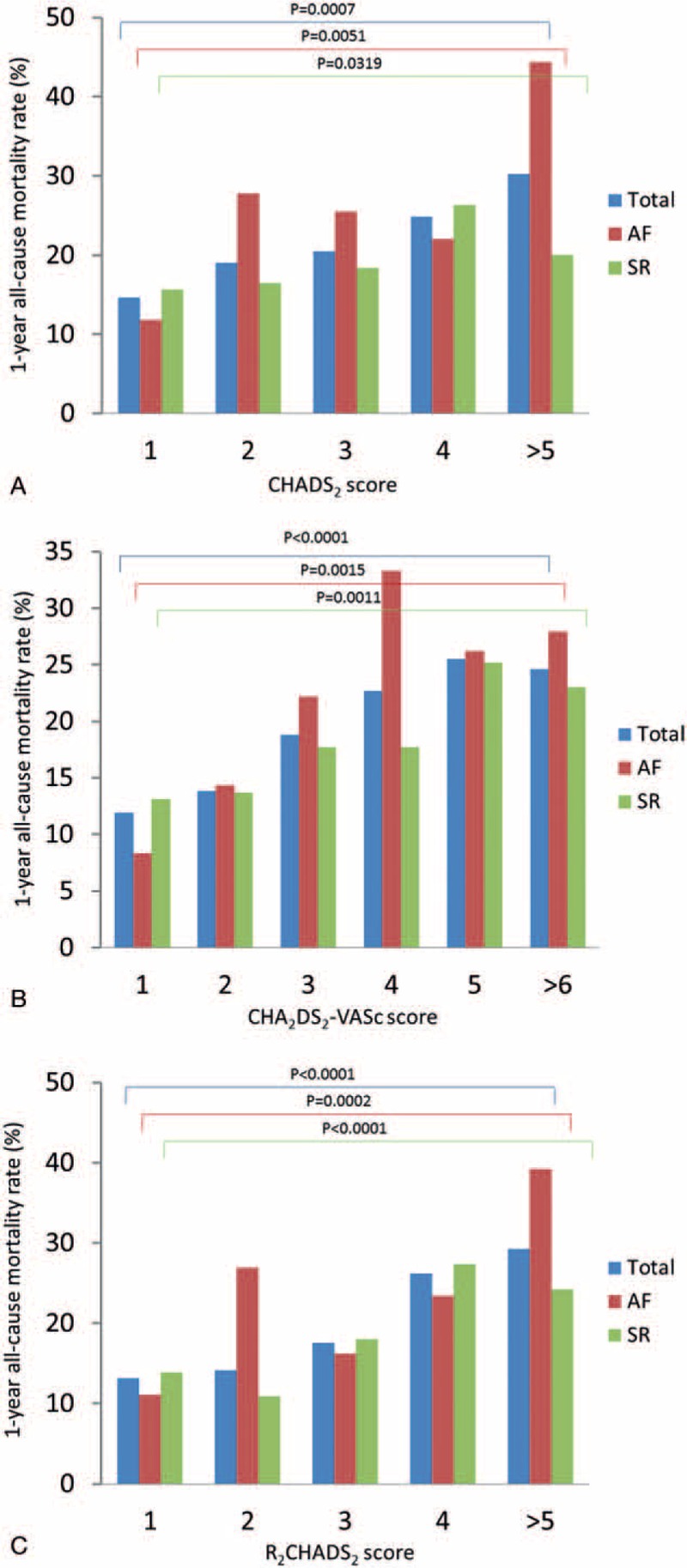
A, One-year all-cause mortality rate among different CHADS_2_ scores in SHF patients with and without AF. SHF patients who had high CHADS_2_ scores had a high 1-year mortality rate regardless of AF or not (all *P* < .05). AF = atrial fibrillation, CHADS2 = congestive heart failure, hypertension, age, diabetes, stroke (doubled), SHF = systolic heart failure, SR = sinus rhythm. B, One-year all-cause mortality rate among different CHA_2_DS_2_-VASc scores in SHF patients with and without AF. SHF patients who had high CHA_2_DS_2_-VASc scores had a high 1-year mortality rate regardless of AF or not (all *P* < .05). AF = atrial fibrillation, CHA_2_DS_2_-VASc = congestive heart failure, hypertension, age ≥ 75 (doubled), diabetes, stroke (doubled), vascular disease, age 65–74, and sex category (female), SHF = systolic heart failure, SR = sinus rhythm. C, One-year all-cause mortality rate among different R_2_CHADS_2_ scores in SHF patients with and without AF. SHF patients who had high R_2_CHADS_2_ scores had a high 1-year mortality rate regardless of AF or not (all *P* < .05). AF = atrial fibrillation, R_2_CHADS_2_ = renal dysfunction (doubled), congestive heart failure, hypertension, age, diabetes, stroke (doubled), SHF = systolic heart failure, SR = sinus rhythm.

**Figure 2:**
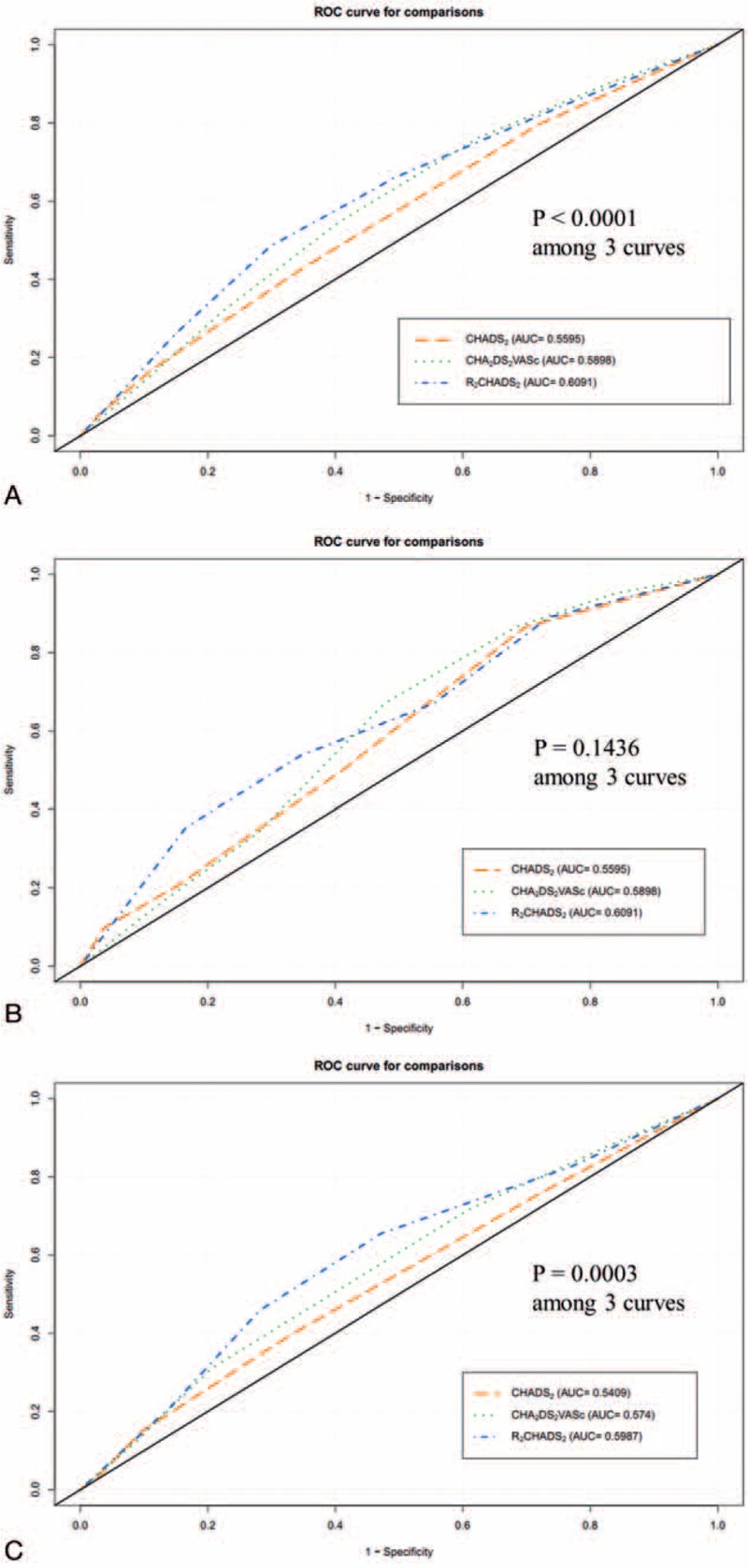
A, The ROC curves for CHADS_2_, CHA_2_DS_2_-VASc, and R_2_CHADS_2_ scoring systems in predicting 1-year all-cause mortality in SHF patients. Based on AUCs for predicting all-cause mortality in SHF patients, CHADS_2_, CHA_2_DS_2_-VASc, and R_2_CHADS_2_ scoring systems had c-indices of 0.5595, 0.5898, and 0.6091, respectively (DeLong test, *P* < .0001). AUC = area under the curve, CHADS_2_ = congestive heart failure, hypertension, age, diabetes, stroke (doubled), CHA_2_DS_2_-VASc = congestive heart failure, hypertension, age ≥ 75 (doubled), diabetes, stroke (doubled), vascular disease, age 65–74, and sex category (female), R_2_CHADS_2_ = renal dysfunction (doubled), congestive heart failure, hypertension, age, diabetes, stroke (doubled), ROC = receiver operating characteristic, SHF = systolic heart failure. B, The ROC curves of CHADS_2_, CHA_2_DS_2_-VASc, and R_2_CHADS_2_ scoring systems for predicting 1-year all-cause mortality in SHF patients with AF. Based on AUCs for predicting all-cause mortality in SHF patients with AF, the CHADS_2_, CHA_2_DS_2_-VASc, and R_2_CHADS_2_ scoring systems had c-indices of 0.5878, 0.6055, and 0.6267, respectively (DeLong test, *P* = .1436). AUC = area under the curve, CHADS_2_ = congestive heart failure, hypertension, age, diabetes, stroke (doubled), CHA_2_DS_2_-VASc = congestive heart failure, hypertension, age ≥ 75 (doubled), diabetes, stroke (doubled), vascular disease, age 65–74, and sex category (female), R_2_CHADS_2_ = renal dysfunction (doubled), congestive heart failure, hypertension, age, diabetes, stroke (doubled), ROC = receiver operating characteristic, SHF = systolic heart failure. C, The ROC curves for CHADS_2_, CHA_2_DS_2_-VASc, and R_2_CHADS_2_ scoring systems used to predict 1-year all-cause mortality in SHF patients without AF. Based on AUCs for predicting all-cause mortality in SHF patients without AF, the CHADS_2_, CHA_2_DS_2_-VASc, and R_2_CHADS_2_ scoring systems had c-indices of 0.5409, 0.5740, and 0.5987, respectively (DeLong test, *P* = .0003). AUC = area under the curve, CHADS_2_ = congestive heart failure, hypertension, age, diabetes, stroke (doubled), CHA_2_DS_2_-VASc = congestive heart failure, hypertension, age ≥ 75 (doubled), diabetes, stroke (doubled), vascular disease, age 65–74, and sex category (female), R_2_CHADS_2_ = renal dysfunction (doubled), congestive heart failure, hypertension, age, diabetes, stroke (doubled), ROC = receiver operating characteristic, SHF = systolic heart failure.

Figure 1 compares 1-year mortality rates in SHF patients with or without AF. The figure shows that, regardless of AF, 1-year mortality in SHF patients had significant positive associations with CHADS_2_, CHA_2_DS_2_-VASc, and R_2_CHADS_2_ scores (all *P* < .05). Figure 2 compares the ROC curves for different risk scoring systems used to predict 1-year mortality. Figure 2A shows that, for predicting totally mortality in SHF patients, the c-indexes based on area under the curve (AUC) were 0.5595 for CHADS_2_, 0.5898 for CHA_2_DS_2_-VASc, and 0.6091for R_2_CHADS_2_. That is, the c-index for R_2_CHADS_2_ score was significantly higher than those for CHADS_2_ and CHA_2_DS_2_-VASc (DeLong test, *P* < .0001). Additionally, the R_2_CHADS_2_ score had a significantly higher NRI in comparison with the CHADS_2_ score (+39.8%; 95% CI: 26.2%–53.3%; *P* < .0001) and in comparisons with the CHA_2_DS_2_-VASc score (+20.5%; 95% CI: 6.8%–34.3%; *P* < .0001). Figure 2B shows that, for predicting all-cause mortality in SHF patients with AF, the c-indexes based on AUC did not significantly differ (*P* = .1436 in DeLong test) among the 3 scoring systems (0.5878, 0.6055, and 0.6267 for CHADS_2_, CHA_2_DS_2_-VASc, and R_2_CHADS_2_, respectively). Figure 2C shows that, for predicting all-cause mortality in SHF patients without AF, the c-index based on AUC was significantly higher (DeLong test, *P* = .0003) for R_2_CHADS_2_ (0.5987) compared with CHADS_2_ (0.5409), and CHA_2_DS_2_-VASc (0.5740). Additionally, the R2CHADS2 score obtained a significantly higher NRI in comparison with CHADS2 (+39.4%; 95% CI: 22.9%–55.8%; *P* < .0001) and in comparison with CHA2DS2-VASc (+27.0%; 95% CI: 10.6%–43.4%; *P* = .0015).

## 4 Discussion

This SHF cohort study had 3 major findings. First, all 3 scoring systems are moderately accurate mortality predictors in SHF patients with or without AF. Second, R_2_CHADS_2_ is the best all-cause mortality predictor in total SHF patients without AF. However, the predictive accuracy does not significantly differ in SHF patients with AF. Third, regardless of the risk factors included in each scoring system, CKD was the only independent predictor of 1-year all-cause mortality in SHF patients with or without AF.

Previous studies show that CHADS_2_ score predicts all-cause mortality in SHF patients who undergo cardiac resynchronization therapy.^[17]^ Lip et al reported that the CHA_2_DS_2_-VASc score was associated with all-cause mortality risk in patients with incidental HF with or without AF. However, the predictive accuracy was modest.^[18]^ Our study showed that, for predicting all-cause mortality in SHF, R_2_CHADS_2_ score is more accurate than CHADS_2_ and CHA_2_DS_2_-VASc. The CKD is an important component of the R_2_CHADS_2_ score. Our study suggested that advanced age, low BMI, and comorbidity of DM, CAD, and PAOD are mortality risk factors in CKD. Previous studies show that renal insufficiency is an independent predictor of all-cause mortality in patients with diastolic or systolic dysfunction, in ambulatory patients with congestive HF, in HF patients (symptomatic or asymptomatic) with left ventricle systolic dysfunction, and in female patients with HF with systolic preserved or depressed systolic function.^[21–24]^ Most (73%) of the HFrEF patients in our study were male, and most were middle aged. CKD, a major component of R_2_CHADS_2_ score, was an independent predictor of all-cause mortality. The findings of this study are generally consistent with the literature despite some differences in patient characteristics. However, no studies have compared the use of CHADS_2_, CHA_2_DS_2_-VASc, and R_2_CHADS_2_ scores for predicting all-cause mortality in SHF patients with or without AF. This cohort study showed that, for predicting all-cause mortality in SHF patients with AF, all 3 scores had moderate accuracy, and predictive accuracy did not significantly differ. However, R_2_CHADS_2_ was the best mortality predictor specifically in those without AF. This study also showed CKD is the only independent predictor of all-cause mortality in SHF patients with or without AF, even when the analysis includes CHADS_2_ or CHA_2_DS_2_-VASc scores. This study confirmed previous reports that BMI, thyroid disorder, and valvular surgery history are independent predictors of all-cause mortality in SHF patients.^[5,25–27]^ In SHF patients without AF, the only 2 independent predictors of all-cause mortality were CKD and BMI whereas CHADS_2_ and CHA_2_DS_2_-VASc were not independent predictors of all-cause mortality. These data may explain why R_2_CHADS_2_ was the superior predictor, especially in SHF without AF. Notably, this study showed that, in addition to these 3 scores, another independent predictor of all-cause mortality in SHF with AF is valvular surgery, which is consistent with previous reports that valvular disease is an independent predictor of all-cause mortality in HF patients with newly diagnosed AF.^[25,28]^ The TSOC-HFrEF registry only enrolled patients with reduced left ventricular function (<40%).^[19]^ During hospitalization, 36.5% of patients used inotropic agents. The use of inotropic agents may explain why physicians did not prescribe ACEI/ARB or beta-blocker at discharge. Nevertheless, the results for the TSOC-HFrEF registry suggest that guideline-directed medical treatment was under-utilized. This study showed that physicians managing HF should implement an evidence-based practice algorithm to improve HF care quality. Nevertheless, this study showed that, compared with 2 other commonly used risk-scoring systems, R_2_CHADS_2_ score is a better predictor of 1-year all-cause mortality in HFrEF patients. The patients with high-risk scores had a high 1-year mortality rate. More aggressive therapeutic management and frequent clinical follow-up may be indicated for these patients.

### 4.1 Study limitations

Some limitations of this study are noted. First, the treatment strategy and the achievement of therapeutic goal of these risk factors (e.g., glycated hemoglobin level in diabetic patients and blood pressure level in hypertensive patients) may influence the impact of these diseases on all-cause mortality. Further prospective studies should be conducted to identify if the treatment of these diseases may influence the outcome of HFrEF patients. Second, this study did not compare other scoring systems such as the MAGGIC HF survival risk score or the Seattle Heart Failure Model. Nevertheless, this prospective study was the first and largest study of HF in Taiwan. Further studies are needed to survey and stratify HF risk specifically in Asian populations. Third, the only exclusion criterion in this study was age less than 18 years old. The percentage of patients with cancer was quite low in this registry (2.9%), and the patients with cancer did not show significantly higher mortality (*P* = .249). Discharge medications, including anticoagulants for AF, were not considered in the mortality analysis to avoid selection bias in discharge medications between mortality and survival (e.g., those with pulmonary edema, shock status, active bleeding, or other severe comorbidities during hospitalization or follow-up may not have had an opportunity for treatment with HF medication or anticoagulants). Therefore, the impact of medications on mortality in this retrospective study of registry data would have been difficult or even impossible. Nevertheless, all these factors such as cancer status and medications might affect mortality in SHF patients. Further prospective studies are needed to address this issue. Fourth, our study only enrolled patients who had heart failure with reduced ejection fraction, 73% of whom were middle-aged (63 ± 16 years old) males. Compared with the cohorts in other heart failure registries and surveys which also included some patients with preserved ejection fraction, our cohort was younger and had a larger percentage of male patients.^[29–31]^ This difference may limit the generalizability of our findings and their applicability to other cohorts.

## 5 Conclusion

The CHADS_2_, CHA_2_DS_2_-VASc, and R_2_CHADS_2_ scores are moderately accurate predictors of all-cause mortality in SHF patients with or without AF. However, only CKD and R_2_CHADS_2_ scores are independent predictors of 1-year all-cause mortality in SHF patients with or without AF. In terms of predicting all-cause mortality in SHF patients, R_2_CHADS_2_ is the best of the three scoring systems, especially in SHF patients without AF.

## Acknowledgments

The TSOC-HFrEF Registry is supported by the Taiwan Society of Cardiology. The 21 medical centers that treated the patients enrolled in this study are listed below in alphabetical order.

Chang Gung Memorial Hospital, Keelung, Taiwan; Chang Gung Memorial Hospital, Linkou, Taiwan; Cheng Hsin General Hospital, Taipei, Taiwan; Chimei Medical Center, Tainan, Taiwan; China Medical University Hospital, Taichung, Taiwan; Chung-Shan Medical University Hospital, Taichung, Taiwan; E-Da Hospital, Kaohsiung, Taiwan; Far Eastern memorial Hospital, New Taipei City, Taiwan; Hualien Tzu Chi Hospital, Buddhist Tzu Chi Medical Foundation, Hualien, Taiwan; Kaohsiung Chang Gung Memorial Hospital, Kaohsiung, Taiwan; Kaohsiung Medical University Chung-Ho Memorial Hospital, Kaohsiung, Taiwan; Kaohsiung Veterans General Hospital, Kaohsiung, Taiwan; MacKay Memorial Hospital, Taipei, Taiwan; National Cheng Kung University Hospital, Tainan, Taiwan; National Taiwan University Hospital, Hsinchu Branch; National Taiwan University Hospital, Taipei, Taiwan; Shin Kong Wu Ho-Su Memorial Hospital, Taipei, Taiwan; Taichung Veterans General Hospital, Taichung, Taiwan; Taipei Tzu Chi Hospital, Buddhist Tzu Chi Medical Foundation, New Taipei City, Taiwan; Taipei Veterans General Hospital, Taipei, Taiwan; Tri-Service General Hospital and National Defense Medical Center, Taipei, Taiwan.

## Supplementary Material

Supplemental Digital Content

## References

[1] Levy WC Mozaffarian D Linker DT The Seattle heart failure model: prediction of survival in heart failure. Circulation 2006;113:1424–33. 16534009 10.1161/CIRCULATIONAHA.105.584102

[2] Pocock SJ Wang D Pfeffer MA Predictors of mortality and morbidity inpatients with chronic heart failure. Eur Heart J 2006;27:65–75. 16219658 10.1093/eurheartj/ehi555

[3] O’Connor CM Hasselblad V Mehta RH Triage after hospitalization with advanced heart failure: the ESCAPE (Evaluation Study of Congestive Heart Failure and Pulmonary Artery Catheterization Effectiveness) risk model and discharge score. J Am Coll Cardiol 2010;55:872–8. 20185037 10.1016/j.jacc.2009.08.083 PMC3835158

[4] Lee DS Austin PC Rouleau JL Predicting mortality among patients hospitalized for heart failure: derivation and validation of a clinical model. JAMA 2003;290:2581–7. 14625335 10.1001/jama.290.19.2581

[5] Pocock SJ Ariti CA McMurray JJ Meta-Analysis Global Group in Chronic Heart Failure. Predicting survival in heart failure: a risk score based on 39 372 patients from 30 studies. Eur Heart J 2013;34:1404–13. 23095984 10.1093/eurheartj/ehs337

[6] Gage BF Waterman AD Shannon W Validation of clinical classification schemes for predicting stroke: results from the National Registry of Atrial Fibrillation. JAMA 2001;285:2864–70. 11401607 10.1001/jama.285.22.2864

[7] Lip GY Nieuwlaat R Pisters R Refining clinical risk stratification for predicting stroke and thromboembolism in atrial fibrillation using a novel risk factor-based approach: the Euroheart survey on atrial fibrillation. Chest 2010;137:263–72. 19762550 10.1378/chest.09-1584

[8] Camm AJ Kirchhof P Lip GY European Heart Rhythm Association; European Association for Cardio-Thoracic Surgery. Guidelines for the management of atrial fibrillation: the task force for the management of atrial fibrillation of the European society of cardiology (ESC). Eur Heart J 2010;3:2369–429. 10.1093/eurheartj/ehq278 20802247

[9] Camm AJ Lip GY De Caterina R ESC Committee for Practice Guidelines (CPG). 2012 focused update of the ESC Guidelines for the management of atrial fibrillation: an update of the 2010 ESC Guidelines for the management of atrial fibrillation. Developed with the special contribution of the European Heart Rhythm Association. Eur Heart J 2012;33:2719–47. 22922413 10.1093/eurheartj/ehs253

[10] Piccini JP Stevens SR Chang Y ROCKET AF Steering Committee and Investigators. Renal dysfunction as a predictor of stroke and systemic embolism in patients with nonvalvular atrial fibrillation: validation of the R (2)CHADS (2) index in the ROCKET AF (Rivaroxaban Once-daily, oral, direct factor Xa inhibition Compared with vitamin K antagonism for prevention of stroke and Embolism Trial in Atrial Fibrillation) and ATRIA (AnTicoagulation and Risk factors In Atrial fibrillation) study cohorts. Circulation 2013;127:224–32. 23212720 10.1161/CIRCULATIONAHA.112.107128

[11] Kornej J Hindricks G Kosiuk J Comparison of CHADS2, R2CHADS2, and CHA2DS2-VASc scores for the prediction of rhythm outcomes after catheterablation of atrial fibrillation: the Leipzig Heart Center AF Ablation Registry. Circ Arrhythm Electrophysiol 2014;7:281–7. 24610790 10.1161/CIRCEP.113.001182

[12] Saliba W Rennert G CHA2DS2-VASc score is directly associated with the risk of pulmonary embolism in patients with atrial fibrillation. Am J Med 2014;127:45–52. 24384101 10.1016/j.amjmed.2013.10.004

[13] Cetin M Cakici M Zencir C Prediction of coronary artery disease severity using CHADS2 and CHA2DS2-VASc scores and a newly defined CHA2DS2-VASc-HS score. Am J Cardiol 2014;113:950–6. 24444782 10.1016/j.amjcard.2013.11.056

[14] Svendsen JH Nielsen JC Darkner S CHADS2 and CHA2DS2-VASc score to assess risk of stroke and death in patients paced for sick sinus syndrome. Heart 2013;99:843–8. 23539553 10.1136/heartjnl-2013-303695 PMC3664372

[15] Mitchell LB Southern DA Galbraith D Prediction of stroke or TIA in patients without atrial fibrillation using CHADS2 and CHA2DS2-VASc scores. Heart 2014;100:1524–30. 24860007 10.1136/heartjnl-2013-305303

[16] Biancari F Asim Mahar MA Kangasniemi OP CHADS2 and CHA2DS2-VASc scores for prediction of immediate and late stroke after coronary artery bypass graft surgery. J Stroke Cerebrovasc Dis 2013;22:1304–11. 23253529 10.1016/j.jstrokecerebrovasdis.2012.11.004

[17] Paoletti Perini A Bartolini S Pieragnoli P CHADS2 and CHA2DS2-VASc scores to predict morbidity and mortality in heart failure patients candidates to cardiac resynchronization therapy. Europace 2014;16:71–80. 23828875 10.1093/europace/eut190

[18] Melgaard L Gorst-Rasmussen A Lane DA Assessment of the CHA2DS2-VASc score in predicting ischemic stroke, thromboembolism, and death in patients with heart failure with and without atrial fibrillation. JAMA 2015;314:1030–8. 26318604 10.1001/jama.2015.10725

[19] Wang CC Chang HY Yin WH TSOC-HFrEF registry: a registry of hospitalized patients with decompensated systolic heart failure: description of population and management. Acta Cardiol Sin 2016;32:400–11. 27471353 10.6515/ACS20160704A PMC4963416

[20] Stevens LA Coresh J Greene T Medical progress-assessing kidney function-measured and estimated glomerular filtration rate. N Engl J Med 2006;354:2473–83. 16760447 10.1056/NEJMra054415

[21] Bibbins-Domingo K Lin F Vittinghoff E Renal insufficiency as an independent predictor of mortality among women with heart failure. J Am Coll Cardiol 2004;44:1593–600. 15489091 10.1016/j.jacc.2004.07.040

[22] McAlister FA Ezekowitz J Tonelli M Renal insufficiency and heart failure: prognostic and therapeutic implications from a prospective cohort study. Circulation 2004;109:1004–9. 14769700 10.1161/01.CIR.0000116764.53225.A9

[23] Mahon NG Blackstone EH Francis GS The prognostic value of estimated creatinine clearance alongside functional capacity in ambulatory patients with chronic congestive heart failure. J Am Coll Cardiol 2002;40:1106–13. 12354436 10.1016/s0735-1097(02)02125-3

[24] Dries DL Exner DV Domanski MJ The prognostic implications of renal insufficiency in asymptomatic and symptomatic patients with left ventricular systolic dysfunction. J Am Coll Cardiol 2000;35:681–9. 10716471 10.1016/s0735-1097(99)00608-7

[25] Ho KK Anderson KM Kannel WB Survival after the onset of congestive heart failure in Framingham Heart Study subjects. Circulation 1993;88:107–15. 8319323 10.1161/01.cir.88.1.107

[26] Mitchell JE Hellkamp AS Mark DB Thyroid function in heart failure and impact on mortality. JACC Heart Fail 2013;1:48–55. 24159562 10.1016/j.jchf.2012.10.004 PMC3803999

[27] Lin GM Li YH Yin WH Taiwan Society of Cardiology (TSOC) Heart Failure with Reduced Ejection Fraction (HFrEF) registry investigators and committee. The obesity-mortality paradox in patients with heart failure in Taiwan and a collaborative meta-analysis for east Asian patients. Am J Cardiol 2016;118:1011–8. 27521221 10.1016/j.amjcard.2016.06.056

[28] Miyasaka Y Barnes ME Bailey KR Mortality trends in patients diagnosed with first atrial fibrillation: a 21-year community-based study. J Am Coll Cardiol 2007;49:986–92. 17336723 10.1016/j.jacc.2006.10.062

[29] Adams KF Jr Fonarow GC Emerman CL ADHERE Scientific Advisory Committee and Investigators. Characteristics and outcomes of patients hospitalized for heart failure in the United States: rationale, design, and preliminary observations from the first 100,000 cases in the Acute Decompensated Heart Failure National Registry (ADHERE). Am Heart J 2005;149:209–16. 15846257 10.1016/j.ahj.2004.08.005

[30] Nieminen MS Brutsaert D Dickstein K EuroHeart Survey Investigators. Heart Failure Association, European Society of Cardiology. EuroHeart Failure Survey II (EHFS II): a survey on hospitalized acute heart failure patients: description of population. Eur Heart J 2006;27:2725–36. 17000631 10.1093/eurheartj/ehl193

[31] Sato N Kajimoto K Keida T TEND Investigators. Clinical features and outcome in hospitalized heart failure in Japan (from the ATTEND Registry). Circ J 2013;77:944–51. 23502987 10.1253/circj.cj-13-0187

