# Supplementary material for: Mortality prediction using CHADS2/CHA2DS2-VASc/R2CHADS2 scores in systolic heart failure patients with or without atrial fibrillation
Source: Medicine (Baltimore). 2017 Oct 27;96(43):e8338. doi: 10.1097/MD.0000000000008338 (PMC5671841; doi:10.1097/MD.0000000000008338)
Supplement: Supplemental Digital Content [file medi-96-e8338-s001.doc]

Supplementary table 1. Baseline characteristics of SHF patients with AF (354 patients)

| Variables | Total  (n=354) | Mortality group  (n=82) | Survival group  (n=272) | P value |
| --- | --- | --- | --- | --- |
| Age | 68.6 ± 14.3 | 71.9 ± 13.1 | 67.6 ± 14.5 | 0.011 |
| Male gender | 254 (71.8%) | 56 (68.3%) | 198 (72.8%) | 0.427 |
| Smoking | 181 (51.1%) | 44 (53.7%) | 137 (50.4%) | 0.601 |
| BMI | 24.8 ± 4.8 | 24.1 ± 4.4 | 25.0 ± 4.9 | 0.149 |
| AF type  Paroxysmal  Non-paroxysmal | 105 (29.7%)  249 (70.3%) | 32 (39%)  50 (61%) | 73 (26.8%)  199 (73.2%) | 0.034 |
| HTN | 107 (30.2%) | 24 (29.3%) | 83 (30.5%) | 0.891 |
| DM | 145 (41%) | 46 (56.1%) | 99 (36.4%) | 0.001 |
| Advanced HF* | 329 (92.9%) | 76 (92.7%) | 253 (93.0%) | 0.918 |
| Dyslipidemia | 55 (15.5%) | 14 (17.1%) | 41 (15.1%) | 0.661 |
| Old stroke | 52 (14.7%) | 15 (18.3%) | 37 (13.6%) | 0.293 |
| Old MI | 83 (23.4%) | 22 (26.8%) | 61 (22.4%) | 0.409 |
| CAD | 143 (40.4%) | 36 (43.9%) | 107 (39.3%) | 0.460 |
| Previous HF admission | 164 (46.3%) | 43 (52.4%) | 121 (44.5%) | 0.205 |
| CKD† | 118 (33.3%) | 40 (48.8%) | 78 (28.7%) | 0.001 |
| PAOD | 21 (5.9%) | 8 (9.8%) | 13 (4.8%) | 0.094 |
| COPD | 56 (15.8%) | 17 (20.7%) | 39 (14.3%) | 0.164 |
| OSA | 15 (4.2%) | 1 (1.2%) | 14 (5.1%) | 0.207 |
| Thyroid disorder | 25 (7.1%) | 9 (11%) | 15 (5.9%) | 0.115 |
| Cancer | 14 (4%) | 5 (6.1%) | 9 (3.3%) | 0.256 |
| Previous  Valvular surgery | 31 (8.8%) | 12 (14.6%) | 19 (7%) | 0.032 |
| Echocardiography‡ |  |  |  |  |
| LA size (mm) | 49.8 ± 9.4 | 51.2 ± 9.5 | 49.3 ± 9.3 | 0.136 |
| LVEF (%) | 28.8 ± 8.3 | 28.0 ± 8.3 | 29.1 ± 8.3 | 0.348 |
| ACEI/ARB | 209 (59.0%) | 36 (43.9%) | 173 (63.6%) | 0.001 |
| Beta-blocker | 189 (53.4%) | 31 (37.8%) | 158 (58.1%) | 0.002 |
| Diuretics | 291 (82.2%) | 58 (70.7%) | 233 (85.7%) | 0.002 |
| CCB | 37 (10.5%) | 6 (7.3%) | 31 (11.4%) | 0.410 |
| Digoxin | 136 (38.4%) | 29 (35.4%) | 107 (39.3%) | 0.605 |
| Antiplatelet | 197 (55.6%) | 29 (35.4%) | 128 (47.1%) | 0.076 |
| Anticoagulation | 152 (42.9%) | 28 (34.1%) | 124 (45.6%) | 0.075 |
| Antiarrhythmia | 70 (19.8%) | 17 (20.7%) | 53 (19.5%) | 0.804 |
| Risk scoring system |  |  |  |  |
| CHADS_2_ score | 2.4 ± 1.1 | 2.7 ± 1.1 | 2.3 ± 1.1 | 0.011 |
| CHA_2_DS_2_-VASc score | 3.6 ± 1.7 | 4.1 ± 1.5 | 3.4 ± 1.7 | 0.001 |
| R_2_CHADS_2_ score | 3.1 ± 1.6 | 3.7 ± 1.7 | 2.9 ± 1.6 | <0.001 |

Data are expressed as means ± SD or % (n).

ACEI/ARB= angiotensin-converting enzyme inhibitors/ angiotensin-receptor blockers ; AF= atrial fibrillation; BMI= body mass index; CAD= coronary artery disease; CCB= calcium channel blocker; CHADS_2_= Congestive heart failure, Hypertension, Age, Diabetes, Stroke (doubled); CHA_2_DS_2_-VASc= Congestive heart failure, Hypertension, Age ≥ 75 (doubled), Diabetes, Stroke (doubled), Vascular disease, Age 65–74, and Sex category (female); CKD= chronic kidney disease; COPD= chronic obstructive pulmonary disease; DM= diabetes mellitus; HF= heart failure; LA= left atrium; LVEF= left ventricular ejection fraction; MI= myocardial infarction; OSA= obstructive sleep apnea; PAOD= peripheral artery occlusion disease; R_2_CHADS_2_= Renal Dysfunction (doubled), Congestive Heart Failure, Hypertension, Age, Diabetes, Stroke (doubled); SHF= systolic heart failure; SD= standard deviation.

* Advanced HF, New York Heart Association functional class ≥3.

† CKD= estimated glomerular filtration rate < 60 ml/min/1.73m^2^.

‡ Data collected during index hospitalization.

Supplementary table 2. Baseline characteristics of SHF patients without AF (957 patients)

| Variables | Total  (n=957) | Mortality group  (n=168) | Survival group  (n=789) | P value |
| --- | --- | --- | --- | --- |
| Age | 61.3 ± 16.4 | 67.1 ± 14.5 | 60.0 ± 16.5 | <0.001 |
| Male gender | 697 (72.8%) | 121 (72.0%) | 576 (73.0%) | 0.795 |
| Smoking | 487 (50.9%) | 78 (46.4%) | 409 (51.8%) | 0.234 |
| BMI | 25.4 ± 5.2 | 23.7 ± 5.5 | 25.8 ± 5.1 | <0.001 |
| HTN | 338 (35.3%) | 52 (31.0%) | 286 (36.2%) | 0.214 |
| DM | 434 (45.4%) | 86 (51.2%) | 348 (44.1%) | 0.094 |
| Advanced HF* | 833 (87.0%) | 152 (90.5%) | 681 (86.3%) | 0.164 |
| Dyslipidemia | 239 (25.0%) | 36 (21.4%) | 203 (25.7%) | 0.280 |
| Old stroke | 72 (7.5%) | 15 (8.9%) | 57 (7.2%) | 0.447 |
| Old MI | 245 (25.6%) | 50 (29.8%) | 195 (24.7%) | 0.174 |
| CAD | 408 (42.6%) | 83 (49.4%) | 325 (41.2%) | 0.051 |
| Previous HF admission | 361 (37.7%) | 65 (38.7%) | 296 (37.5%) | 0.775 |
| CKD† | 291 (30.4%) | 79 (47.0%) | 212 (26.9%) | <0.001 |
| PAOD | 67 (7.0%) | 19 (11.3%) | 48 (6.1%) | 0.016 |
| COPD | 83 (8.7%) | 23 (13.7%) | 60 (7.6%) | 0.011 |
| OSA | 21 (2.2%) | 3 (1.8%) | 18 (2.3%) | 1.000 |
| Thyroid disorder | 38 (4.0%) | 12 (7.1%) | 26 (3.3%) | 0.020 |
| Cancer | 24 (2.5%) | 5 (3.0%) | 19 (2.4%) | 0.669 |
| Previous  Valvular surgery | 32 (3.3%) | 8 (4.8%) | 24 (3.0%) | 0.260 |
| Echocardiography‡ |  |  |  |  |
| LA size (mm) | 45.1 ± 8.2 | 44.5 ± 8.2 | 45.2 ± 8.0 | 0.339 |
| LVEF (%) | 28.4 ± 9.0 | 28.0 ± 9.2 | 28.5 ± 8.9 | 0.546 |
| Medication |  |  |  |  |
| ACEI/ARB | 569 (59.5%) | 68 (40.5%) | 501 (63.5%) | <0.001 |
| Beta-blocker | 572 (59.8%) | 74 (44.0%) | 498 (63.1%) | <0.001 |
| Diuretics | 743 (77.6%) | 109 (64.9%) | 634 (80.4%) | <0.001 |
| CCB | 115 (12.0%) | 18 (10.7%) | 97 (12.3%) | 0.695 |
| Digoxin | 198 (20.7%) | 41 (24.4%) | 157 (19.9%) | 0.190 |
| Antiplatelet | 593 (62.0%) | 97 (57.7%) | 496 (62.9%) | 0.214 |
| Anticoagulation | 126 (13.2%) | 14 (8.3%) | 112 (14.2%) | 0.044 |
| Antiarrhythmia | 128 (13.4%) | 25 (14.9%) | 103 (13.1%) | 0.528 |
| Risk scoring system |  |  |  |  |
| CHADS_2_ score | 2.2 ± 1.0 | 2.4 ± 1.1 | 2.2 ± 1.0 | 0.068 |
| CHA_2_DS_2_-VASc score | 3.2 ± 1.6 | 3.6 ± 1.7 | 3.1 ± 1.6 | 0.002 |
| R_2_CHADS_2_ score | 2.8 ± 1.5 | 3.3 ± 1.6 | 2.7 ± 1.4 | <0.001 |

Data are expressed as means ± SD or % (n).

ACEI/ARB= angiotensin-converting enzyme inhibitors/ angiotensin-receptor blockers ; AF= atrial fibrillation; BMI= body mass index; CAD= coronary artery disease; CCB= calcium channel blocker; CHADS_2_= Congestive heart failure, Hypertension, Age, Diabetes, Stroke (doubled); CHA_2_DS_2_-VASc= Congestive heart failure, Hypertension, Age ≥ 75 (doubled), Diabetes, Stroke (doubled), Vascular disease, Age 65–74, and Sex category (female); CKD= chronic kidney disease; COPD= chronic obstructive pulmonary disease; DM= diabetes mellitus;HF= heart failure; LA= left atrium; LVEF= left ventricular ejection fraction; MI= myocardial infarction; OSA= obstructive sleep apnea; PAOD= peripheral artery occlusion disease; R_2_CHADS_2_= Renal Dysfunction (doubled), Congestive Heart Failure, Hypertension, Age, Diabetes, Stroke (doubled); SHF= systolic heart failure; SD= standard deviation.

* Advanced HF, New York Heart Association functional class ≥3.

† CKD= estimated glomerular filtration rate < 60 ml/min/1.73m^2^.

‡ Data collected during index hospitalization.

Supplementary table 3. Correlation between chronic kidney disease and other baseline characteristics of 1311 SHF Patients

| Variables | R | p value |
| --- | --- | --- |
| Age | 0.199 | <0.001 |
| Male gender | -0.014 | 0.623 |
| Smoking | -0.026 | 0.346 |
| BMI | -0.066 | 0.018 |
| AF | 0.028 | 0.310 |
| HTN | 0.032 | 0.249 |
| DM | 0.207 | <0.001 |
| Advanced HF* | 0.101 | <0.001 |
| Dyslipidemia | 0.029 | 0.299 |
| Old stroke | 0.075 | 0.007 |
| Old MI | 0.128 | <0.001 |
| CAD | 0.154 | <0.001 |
| Previous HF admission | 0.071 | 0.010 |
| PAOD | 0.208 | <0.001 |
| COPD | 0.035 | 0.199 |
| OSA | -0.002 | 0.933 |
| Thyroid disorder | -0.005 | 0.805 |
| Cancer | 0.001 | 0.959 |
| Previous  Valvular surgery | 0.033 | 0.226 |
| Echocardiography‡ |  |  |
| LA size (mm) | -0.018 | 0.542 |
| LVEF (%) | 0.099 | <0.001 |

Data are expressed as means ± SD or % (n).

AF= atrial fibrillation; BMI= body mass index; CAD= coronary artery disease; CKD= chronic kidney disease; COPD= chronic obstructive pulmonary disease; DM= diabetes mellitus; HF= heart failure; LA= left atrium; LVEF= left ventricular ejection fraction; MI= myocardial infarction; OSA= obstructive sleep apnea; PAOD= peripheral artery occlusion disease; SHF= systolic heart failure; SD= standard deviation.

* Advanced HF, New York Heart Association functional class ≥3.

† CKD= estimated glomerular filtration rate < 60 ml/min/1.73m^2^.

‡ Data collected during index hospitalization.
